# Non-Coding RNAs as Key Regulators of Glutaminolysis in Cancer

**DOI:** 10.3390/ijms21082872

**Published:** 2020-04-20

**Authors:** Yunuen Ortiz-Pedraza, J. Omar Muñoz-Bello, Leslie Olmedo-Nieva, Adriana Contreras-Paredes, Imelda Martínez-Ramírez, Elizabeth Langley, Marcela Lizano

**Affiliations:** 1 Unidad de Investigación Biomédica en Cáncer, Instituto Nacional de Cancerología-Instituto de Investigaciones Biomédicas, Universidad Nacional Autónoma de México, Mexico City 14080, Mexico; 2 Posgrado en Biología Experimental, DCBS, Universidad Autónoma Metropolitana-Iztapalapa, Mexico City 09340, Mexico; 3 Departamento de Ciencias de la Salud, Universidad Autónoma Metropolitana-Iztapalapa, Mexico City 09340, Mexico; 4 Departamento de Farmacobiología, Centro de Investigación y Estudios Avanzados del Instituto Politécnico Nacional, Sede sur, Mexico City 14330, Mexico; 5 Programa de Doctorado en Ciencias Bioquímicas, Universidad Nacional Autónoma de México, Ciudad Universitaria, Mexico City 04510, Mexico; 6 Departamento de Medicina Genómica y Toxicología Ambiental, Instituto de Investigaciones Biomédicas, Universidad Nacional Autónoma de México, Ciudad Universitaria, Mexico City 04510, Mexico

**Keywords:** cancer, glutaminolysis, miRNAs, lncRNAs

## Abstract

Cancer cells exhibit exacerbated metabolic activity to maintain their accelerated proliferation and microenvironmental adaptation in order to survive under nutrient-deficient conditions. Tumors display an increase in glycolysis, glutaminolysis and fatty acid biosynthesis, which provide their energy source. Glutamine is critical for fundamental cellular processes, where intermediate metabolites produced through glutaminolysis are necessary for the maintenance of mitochondrial metabolism. These include antioxidants to remove reactive oxygen species, and the generation of the nonessential amino acids, purines, pyrimidines and fatty acids required for cellular replication and the activation of cell signaling. Some cancer cells are highly dependent on glutamine consumption since its catabolism provides an anaplerotic pathway to feed the Krebs cycle. Intermediate members of the glutaminolysis pathway have been found to be deregulated in several types of cancers and have been proposed as therapeutic targets and prognostic biomarkers. This review summarizes the main players in the glutaminolysis pathway, how they have been found to be deregulated in cancer and their implications for cancer maintenance. Furthermore, non-coding RNAs are now recognized as new participants in the regulation of glutaminolysis; therefore, their involvement in glutamine metabolism in cancer is discussed in detail.

## 1. Introduction

Glucose and glutamine are the main nutrient sources supporting biosynthesis in mammalian cells. Their catabolism provides cells with adenosine triphosphate (ATP) and the building blocks for macromolecular synthesis that cells need to grow and survive; these are, mainly, nucleic acids, proteins, carbohydrates and lipids. Metabolic reprogramming has been identified as a hallmark of cancer, consisting of increased aerobic glycolysis, glutaminolysis and fatty acid biosynthesis, which affect energy generation in the mitochondria. Long ago, Otto Warburg described how cancer cells showed an increased glucose consumption in relation to normal differentiated tissues. Now we know that glutamine metabolism is as important as glucose metabolism for the production of macromolecules in cancer [1].

Glutamine is an abundant amino acid involved in energy production, homeostasis in pro/antioxidant species and the activation of signaling pathways in cancer. In addition to the antioxidant glutathione (GSH), nucleotides, lipids and amino acids are formed from glutamine metabolism. All of these are needed for many metabolic functions such as growth, proliferation, cell survival and defense against oxidative stress [2]. Glutamine contributes, with reduced nitrogen, to the de novo biosynthesis of diverse nitrogen-containing compounds, such as purine and pyrimidine nucleotides, glucosamine-6-phosphate and nonessential amino acids.

As glutamine is the most abundant amino acid in the blood and muscle tissue, normal proliferating cells use glutamine metabolism as an energy source, mediated by its catabolic products, glutamate and α-ketoglutarate (α-kG), the latter being an intermediate of the tricarboxylic acid cycle (TCA) or Krebs cycle [3]. In 1950, Harry Eagle observed that HeLa tumor cells require an excess of glutamine, in relation to other amino acids in the culture medium, for optimal growth. Furthermore, it has been reported that tumors consume glutamine faster than the surrounding normal tissue [4]. Moreover, most cancer cells are dependent on glutamine being unable to survive under glutamine starvation, an effect that has been termed glutamine addiction [5]. Various types of cancers are highly dependent on glutamine, such as non-small cell lung cancer, breast cancer and brain tumors [6]. Therefore, glutaminolysis has emerged as an important subject of study in order to find therapeutic strategies to combat cancer.

Glutaminolysis is the metabolic pathway in which glutamine is processed into the metabolites that feed the TCA cycle, through a series of enzymes. First, glutaminase isozymes glutaminase 1 and glutaminase 2 (GLS, GLS2) convert glutamine to glutamate, which is a substrate in the synthesis of nucleic acids and amino acids, such as serine. Next, through glutamate dehydrogenase or transaminases, glutamate is converted to α-kG, providing an anaplerotic pathway to TCA. Glutamate is also a substrate for dioxygenases, which are modifiers of proteins and DNA, such as prolyl hydroxylases and histone demethylases, making glutamate a main component of cell signaling and the epigenetic network [7].

In order to satisfy cellular energetic demands while maintaining homeostasis, a large number of energetic sensors and metabolic signaling pathways act coordinately, influencing growth, proliferation and death. Cancer cells disrupt regulated homeostasis to acquire the rapid biosynthesis of ATP and macromolecules, causing an increase in aerobic glycolysis, glutaminolysis and lipid metabolism [8,9]. Among the regulators of metabolic homeostasis, some non-coding RNAs have been recently identified so far [10], including microRNAs (miRNAs) [11] long non-coding RNAs (lncRNAs) [12] and circular non-coding RNAs (circRNAs) [13].

This review focuses primarily on the glutaminolysis pathway and the evidence for the role of miRNAs and lncRNAs in the regulation of this metabolic hallmark in cancer cells.

## 2. Glutamine Metabolism

**Figure 1:**
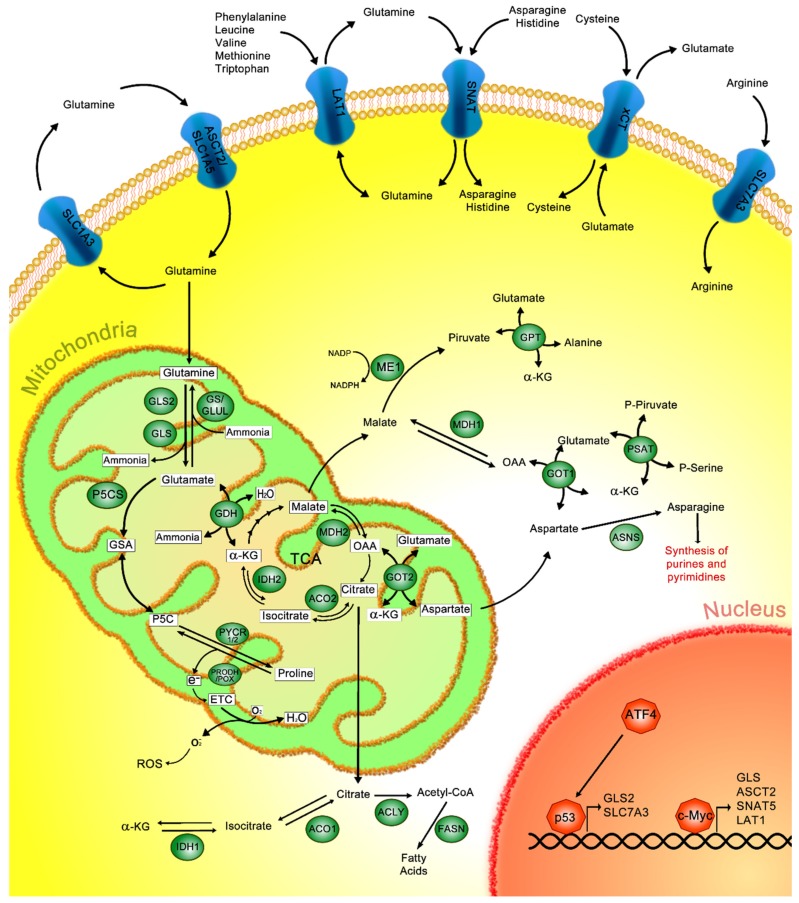
Canonical glutaminolysis pathway. Glutamine is captured in the outer cell membrane through different amino acid transporters such as solute carrier (SLC)1A3, alanine, serine, cysteine, and glutamate transporter (ASCT2/SLC1A5), L-type amino acid transporter 1 (LAT1), sodium-neutral amino acid transporters (SNATs), sodium-independent cysteine–glutamate antiporter (xCT) or SLC7A3. Once in the mitochondria, glutamine is converted to glutamate through glutaminase 1 and glutaminase 2 (GLS, GLS2); inversely, glutamine synthetase (GS) can generate glutamine from glutamate. Glutamate dehydrogenase (GDH) catalyzes the conversion of glutamate to α-ketoglutarate (α-kG) and ammonia. Additionally, α-kG can also be obtained from the isocitrate that is derived from the tricarboxylic acid cycle (TCA); this reaction is catalyzed by isocitrate dehydrogenase 2 (IDH2), and isocitrate is previously obtained from citrate by the activity of aconitase 2 (ACO2). In the cytoplasm, isocitrate dehydrogenase 1 (IDH1) mediates the conversion of α-kG to isocitrate, then isocitrate is transformed into citrate through aconitase 1 (ACO1), which, in turn, is converted to acetyl-coenzyme A (acetyl-CoA) via adenosine triphosphate (ATP) citrate lyase (ACLY), finally producing fatty acids through the activity of fatty acid synthase (FASN). Moreover, α-kG is generated from oxaloacetate (OAA) by glutamate–oxaloacetate transaminases 1 and 2 (GOT1 and GOT2) in the cytoplasm and mitochondria, respectively. Then, cytoplasmic OAA is converted to malate by malate dehydrogenase 1 (MDH1), and further to pyruvate and nicotinamide adenine dinucleotide phosphate (NADPH) by malic enzyme 1 (ME1). Meanwhile, mitochondrial OAA is formed from malate by malate dehydrogenase 2 (MDH2). Additionally, glutamate is transformed to glutamic-γ-semialdehyde (GSA) by delta-1-pyrroline-5-carboxylate synthase (P5CS), which is interconverted to pyrroline-5-carboxylate (P5C) and turned into proline through pyrroline-5-carboxylate reductase (PYCR); conversely, proline is oxidized to P5C by proline–dehydrogenase/proline–oxidase (PRODH/POX) impacting the production of H_2_O or O^−^_2_. Moreover, p53 and c-Myc promote the transcription of several proteins related to glutamine metabolism.

Glutaminolysis is the process that encompasses glutamine uptake to its catabolism [3] (Figure 1). Glutamine plays an important role in normal cell metabolism, functioning as an important nitrogen and carbon donor for the synthesis of purines, pyrimidines and non-essential amino acids [14]. Moreover, glutamine is a precursor of the antioxidant glutathione, promoting the restoration of reduced GSH [15].

Under normal conditions, glutamine enters from the extracellular space via the solute carrier (SLC) group of transporters, including the SLC1, SLC6, SLC7 and SLC38 members, which import glutamine and other amino acids [16]. The excitatory amino acid transporter (EAAT1-5), also known as the alanine, serine, cysteine, and glutamate transporter (ASCT2) belongs to the SLC1 family of transporters and mediates the absorption of neutral amino acids, such as glutamine. ASCT2 is considered to be one of the primary importers of glutamine on the cell surface [17,18,19] (Figure 1).

L-type amino acid transporter 1 (LAT1) belongs to the SLC7 transporter family and functions as a glutamine transporter. This transporter imports leucine, valine, methionine, tryptophan and phenylalanine, which are essential amino acids, in exchange for glutamine [20,21]. Additionally, the sodium-independent cysteine–glutamate antiporter (xCT), which belongs to same SLC7 family, imports cysteine into the cells in exchange for intracellular glutamate [22,23] (Figure 1). It has been previously described that the xCT promotes glutathione biosynthesis by protecting cells from oxidative stress due to cancer [23].

Regarding the SLC38 family, a number of sodium-neutral amino acid transporters (SNATs) have been characterized (Figure 1). These transporters are divided into two groups; the first one comprises the system A amino acid transporters and the second one comprises the system N amino acid transporters. The system A amino acid transporters are SNAT1 (SLC38A1), SNAT2 (SLC38A2) and SNAT4 (SLC38A4), which are Na+-neutral amino acid co-transporters. Conversely, the system N amino acid transporters, SNAT3 (SLC38A1), SNAT5 (SLC38A5) and SNAT8 (SLC38A8), import glutamine, asparagine and histidine amino acids [24,25]. These transporters are implicated in glutamine entrance, triggering the glutaminolysis pathway.

After the absorbed glutamine reaches the mitochondria, glutaminase 1 and glutaminase 2 (GLS, GLS2) enzymes catalyze the formation of glutamate and ammonia from glutamine. Afterwards, glutamate dehydrogenase (GDH) catalyzes the conversion of glutamate to α-kG and ammonia, which are necessary for feeding the TCA and ammonia cycles, respectively. In the TCA cycle, α-kG is obtained from isocitrate through isocitrate dehydrogenase 2 (IDH2) and isocitrate is formed from citrate by aconitase 2 (ACO2). Those reactions take place within the mitochondria. Additionally, in the cytoplasm, α-kG can be converted to isocitrate through isocitrate dehydrogenase 1 (IDH1) and then to citrate though aconitase 1 (ACO1); then, ATP citrate lyase (ACLY) generates acetyl-coenzyme A (acetyl-CoA), which is necessary for fatty acid production via fatty acid synthase (FASN) [3,26,27,28,29].

Furthermore, other mechanisms are proposed for α-kG generation, involving the catalysis of glutamate through the action of glutamate–pyruvate transaminase (GPT) and phosphoserine transaminase (PSAT). Additionally, α-kG is also generated from oxaloacetate (OAA); this reaction is catalyzed by glutamate–oxaloacetate transaminases 1 and 2 (GOT1/2), localized in the cytosol and mitochondria, respectively [28]. Furthermore, transaminases are involved in generating additional nonessential amino acids such as aspartate, alanine, and phosphoserine. For instance, asparagine, derived from aspartate, is necessary for the synthesis of purines and pyrimidines [3,28] (Figure 1).

Products generated by the glutaminolysis pathway are essential to feed the urea cycle and to generate nicotinamide adenine dinucleotide phosphate (NADPH), pyruvate, and amino acids such as proline, which are necessary for cell maintenance. In the mitochondria, glutamate can be metabolized through delta-1-pyrroline-5-carboxylate synthase (P5CS) to generate glutamic-γ-semialdehyde (GSA), which can spontaneously interconvert into its tautomer pyrroline-5-carboxylate (P5C), serving as substrate of pyrroline-5-carboxylate reductase (PYCR) 1/2, which finally generates proline. Proline is oxidized to P5C via proline–dehydrogenase/proline–oxidase (PRODH/POX) activity; this reaction produces electrons that are transferred to the electron transport chain (ETC) to: (1) oxidized O_2_ in order to produce H_2_O; or (2) oxygen, inducing its reduction to form superoxide (O^−^_2_), a known reactive oxygen species (ROS). Furthermore, ROS can participate in the regulation of different signaling pathways and epigenetic mechanisms. In cancer, it has been shown that enzymes involved in proline biosynthesis are dysregulated, promoting an increase in proline, which impacts upon exacerbated tumor growth [30,31,32,33,34].

The mitochondrial malate–aspartate shuttle is characterized by being a transamination–redox transport cycle that mediates the export and import of aspartate/glutamate and malate/α-kG through specific antiporters (I and II, respectively), from the mitochondria to the cytosol. Antiporter I is responsible for maintaining the outflow of aspartate from the mitochondria by allowing glutamate to enter. The exchange of malate and α-kG through antiporter II is necessary for maintaining the TCA cycle, as well as the glutaminolysis pathway [35,36].

In the cytoplasm, aspartate plus α-kG are converted to OAA and glutamate through GOT1; then, OAA is catalyzed to malate by malate dehydrogenase 1 (MDH1) [35]. Cytoplasmic malate is catalyzed to pyruvate and NADPH by the activity of malic enzyme 1 (ME1). Conversely, antiporter II can import malate to the mitochondria, where malate dehydrogenase 2 (MDH2) converts malate to OAA. In the cytoplasm, aspartate is used for the synthesis of amino acids, such as asparagine, synthesized through asparagine synthetase (ASNS). Asparagine can then be used in the Urea cycle and/or in pathways related to nucleotide synthesis [3,28] (Figure 1).

Although glutamine is obtained from the diet, there are other mechanisms by which glutamine can be generated within cells from other metabolic intermediaries. For example, glutamine synthetase (GS), also called glutamate ammonia ligase (GLUL), catalyzes the conversion of glutamate to glutamine. GS is an ATP-dependent enzyme involved in tumor growth and proliferation [37].

Glutamine is a non-essential amino acid, but when cells are deprived of glutamine, GS induces glutamine synthesis; therefore, in this case, glutamine is considered an essential amino acid. Some cancer cells synthesize de novo glutamine through the action of GS, supporting essential processes, such as protein synthesis [38].

The elements involved in the regulation of glutaminolysis are depicted in Figure 1.

## 3. Glutamine Metabolism in Cancer

Several members of the glutaminolysis pathway, including glutamine transporters, enzymes and metabolites, have been found to be altered in many types of cancer, either in function or expression levels. Metabolites obtained from glutaminolysis are key players mediating metabolic reprogramming in cancer and participating in its establishment and development.

A clinicopathological study revealed that ASCT2 and xCT transporters are overexpressed in tongue cancer samples and exhibit a high association with poor prognosis and tumor progression [39]. The molecular mechanisms of ASCT2 were further studied in different kinds of tumors, including melanoma, myeloid leukemia, breast, lung and prostate cancer. When ASCT2 was blocked, using siRNA knockdown or chemical compounds such as Benzylserin and g-LGlutamyl-p-Nitroanilide (GPNA), a reduction in glutamine uptake was observed and, consequently, tumor cell proliferation decreased [40,41,42,43,44]. Moreover, LAT1 overexpression has been observed in non-small cell lung cancer (NSCLC) cells, where LAT1 inhibitor, 2-aminobicyclo-(2,2,1)-heptane-2-carboxylic acid (BCH), promotes a reduction in cell viability [45].

Bröer et al. (2016) [25] reported that in, the HeLa cell line and 143B osteosarcoma cells, the glutamine transporters SNAT1, SNAT2, SNAT4, LAT1 and ASCT2 are highly expressed. When ASCT2 was deleted, glutamine uptake was found to be mediated mainly through SNAT1 and SNAT2 transporters, without affecting cell growth. In contrast, ASCT2 silencing induced apoptosis in hepatoma cells and decreased growth in melanoma and pancreatic cancer cells.

The first enzymes participating in the glutaminolysis pathway are GLS and GLS2, which exert different functions depending on the isoform. GLS has been correlated with tumor growth and malignant phenotypes, being regulated by c-Myc oncoprotein, which increases GLS expression and glutamine uptake [46,47,48]. Additionally, Kamarajan et al. (2017) [49] reported that GLS is overexpressed in primary and metastatic head and neck squamous cell carcinoma (HNSSC) tissues and negatively correlates with disease-free periods.

GLS2 is transcriptionally regulated by p53, but its role in cancer remains unclear. It has been proposed as a tumor suppressor in hepatocellular carcinoma (HCC), where the loss of GLS2 is associated with tumor growth. GLS2 restoration in HCC cells negatively regulates phosphatidylinositol 3-kinase (PI3K/AKT) signaling, promoting the inhibition of migration, invasion and metastasis, and reducing the size of HCC xenograft tumors [50]. In contrast, other studies demonstrated the oncogenic activity of GLS2, where its overexpression is associated with poor overall survival in blood, colorectal, ovarian and thymoma cancers [51].

c-Myc promotes growth and proliferation and is also involved in glutamine metabolism, being selectively bound to ASCT2 and SNAT5 promoter regions, producing the overexpression of those transporters [52]. Additionally, c-Myc induces *SLC7A5* (LAT1), *GLUL* (GS) and *GLS* transcription [46,53,54]. Additionally, c-Myc conditional–transgenic mouse models, which overexpress c-Myc in the liver and kidneys, cause the formation of tumors that overexpress GLS (relative to surrounding tissue) [47,55].

Another transcriptional factor found commonly altered in different types of cancer is p53, which is also related to glutamine metabolism regulation. Using either a model of lymphoma cells with mutated p53 or xenograft tumors with p53 knocked out in colon cancer cells, resistance to glutamine deprivation was observed compared to those models harboring wild type p53. Furthermore, it was shown that, under glutamine deprivation, mutated p53 induced cell cycle arrest in the G1/S phase through p21 expression [56].

Previously, it was demonstrated that p53 regulates the expression of *SLC1A3* (aspartate–glutamate transporter) in HCT116 colon cancer cells. Interestingly, in glutamine deprivation, cancer cells use aspartate to maintain their normal metabolism through the production of glutamate, glutamine, and nucleotide synthesis to rescue cell viability, contributing to cell adaptation to metabolic stress. Meanwhile, in the absence of glutamine, a reduction in proliferation was observed in p53 non-expressing HTC116 cells. Moreover, in a p53-null xenograft model, the failure of TCA-cycle activity was observed in response to glutaminase inhibition, suggesting that p53 helps to maintain the glutaminolysis pathway [57].

Similarly, an in vitro model using mouse embryonic fibroblasts (MEFs) demonstrated that, under glutamine starvation, Activating Transcriptor 4 (ATF4) induces the activation of p53 and, as a consequence, SLC7A3 is expressed. This event promoted high arginine levels inside the cell, causing mTOR activation [58].

The exchange of glutamine with essential amino acids stimulates some signaling pathways, which support cell growth and proliferation. For instance, mammalian target of rapamycin 1 (mTORC1) is activated by glutamine, stimulating protein synthesis [59]. mTORC is a master regulator of cell growth, as well as an inhibitor of apoptosis and autophagy. This activation is probably due to the production of α-kG induced by glutamine plus leucine, which stimulates the lysosomal translocation and activation of mTORC1 in a RagB GTPase-dependent manner [60]. RagB GTPase forms heterodimers, which are anchored to the lysosomal surface membrane. Through unknown mechanisms, the addition of amino acids induces the activation of RagB, leading to the recruitment of mTORC1 to the lysosome [61]. Once in the lysosome, mTORC1 is activated through another GTPase named Rheb [62].

## 4. Therapeutic Approaches Targeting the Glutaminolysis Pathway in Cancer

Since glutaminolysis is necessary for the regulation of signaling pathways related to malignant processes, it is an attractive therapeutic target against cancer. Therefore, various strategies for inhibiting glutaminolysis have been considered.

In a mouse model of HNSCC, it was shown that the inhibition of GLS by bis-2-(5-phenylacetamido-1,3,4-thiadiazol-2-yl) ethyl sulfide (BPTES) leads to apoptosis and caused the inhibition of HNSCC tumor growth, when injected intraperitoneally [63]. Similarly, in orthotopically transplanted mice with human pancreatic tumor cells treated with BPTES nanoparticle (BPTES-NP) therapy, a reduction in GLS activity and tumor growth was observed [64].

Another compound similar to BPTES is Telaglenastat (CB-839), which belongs to the benzo(a) phenanthridinone family. Interestingly, in triple negative breast cancer, the effect induced by CB-839 was significantly more powerful than that exerted by BPTES. The effect of these two inhibitors is achieved through the inhibition of GLS, targeting its allosteric site, thereby regulating the enzymatic activity of GLS and its splice isoforms kidney-type glutaminase (KGA) and glutaminase C (GAC) [65].

Recently, it was shown that in glutamine-dependent cells, such as osteosarcoma (OS) derived cells, treatment with glutamine inhibitors including CB-839, compound 968 and BTES did not inhibit cell proliferation in in vivo pharmacologically achievable concentrations. However, when cells were treated with high doses of such inhibitors, only CB-839 and compound 968 exhibited an inhibitory effect on cell growth. It is known that metformin affects metabolism in tumors through its interaction with mitochondrial complex I. Therefore, when a combination of metformin and CB-839 was applied, in glutamine-deprived media, a reduction was observed in the cell growth of osteosarcoma cell lines. Interestingly, the metabolic profiles showed a decrease in glutamate, aspartate and GSH levels and an increase in glutamine. Additionally, in mice models treated with CB-839 plus metformin, the metastasis rate was inhibited after treatment, indicating that this therapy could be directed toward patients with late stages of osteosarcoma [66].

Furthermore, glutamine flux and metabolism has been measured in cancer patients. Glutamine, labeled as ^18^F-(2S,4R)-4-fluoroglutamine (^18^F-FGln), is mainly transported by ASCT2. In order to determine whether glutamine metabolism could be affected after inhibitor administration, five cancer patients were treated with ^18^F-FGln and measured with dynamic positron emission tomography (PET). Interestingly, it was shown that CB-839 and Sapanisertib (TAK-228), inhibitor of mTORC1/2, therapies decreased the rate of glutaminolysis. Therefore, ^18^F-FGln dynamic PET allows for the study of glutamine flux in patients with cancer, which necessary in order to evaluate specific cancer types with glutamine addiction, as well as responses to targeted therapies with inhibitors of the glutaminolysis pathway [67].

The SLC1A5 receptor was previously identified as the major glutamine transporter, modulating cell growth and oxidative stress in NSCLC. Patients with increased expression of SCL1A5 exhibited shorter overall survival, indicating that it could be an excellent candidate as a prognostic biomarker. NSCLC cells overexpressing SLC1A5 that were treated with the GLS inhibitor GPNA showed inhibition of cell growth, increased autophagy and apoptosis, and a marked reduction in glutamine consumption. Consistent with these results, when SLC1A5 was knocked down genetically or with GPNA, the attenuation of tumor growth was observed in tumor xenografts in mice, demonstrating that SLC1A5 can be considered a therapeutic candidate for NSCLC [68].

V-9302 can block the ASCT2-mediated transport that inhibits glutamine uptake and has been considered as a targeted therapy in tumor cells, since it attenuates proliferation, increasing oxidative stress and cell death, and shows better potency and selectivity than GPNA [69]. Remarkably, Luo et al. (2020) [70] used the polymer 2-deoxyglucose prodrug-based micellar carrier (POEG-p-2DG) that can self-assemble to form micelles that co-deliver V9302 (V9302/POEG-p-2DG) in an aggressive murine breast cancer model, showing a decrease in glutamine uptake and a better antitumor efficacy than V-9302 alone [70]. These results encourage the study of alternative delivery mechanisms of glutaminolysis inhibitors. Further studies are necessary to test the use of nanoparticles, liposomes and monoclonal antibodies as pharmacological carriers for the treatment of glutamine-dependent cancers.

Therapeutic approaches against metabolic reprogramming in cancer should consider not only including compounds that target proteins as described above, but also biomolecules such as ncRNAs, which have recently been described as regulators of metabolism in cancer cells, particularly those related to the metabolism of glutamine. Therefore, further studies are required to explore the potential of ncRNAs as therapeutic targets.

## 5. Non-Coding RNA (ncRNA)

Whole human genome studies, with the help of new technologies, such as next-generation deep sequencing, have shown that less than 10% of the human genome is transcribed. However, only 1–2% of the transcribed RNA is protein-encoding, the rest is called non-coding RNA (ncRNA), which represents most of the RNA contained in a cell. There are more than 10,000 ncRNA transcripts, including short and long non-coding RNA [71,72,73]. Until now, for many ncRNAs, the exact functional mechanisms have been unknown, but evidence suggests that this type of nucleic acid has an important impact on the regulation of various biological processes, such as metabolic homoeostasis, and have been implicated in different diseases such as cancer [10,73,74].

Although the ncRNAs do not encode for proteins, they frequently harbor a poly-A tail in their sequence and they can also be spliced. The ncRNAs are grouped, according to their size, into two classes: (1) small ncRNAs, harboring less than 200 nt, comprising microRNAs (miRNAs), small interfering RNAs (siRNAs), piwi-interacting RNAs (piRNAs), small nucleolar RNAs (snoRNAs), and tRNA-derived fragments (tRFs) [75,76,77] and (2) long non-coding RNAs (lncRNAs) that are more than 200 nt in length and are classified, according to their biogenesis, into intronic, enhancer, promoter, antisense, sense, intergenic and bidirectional lncRNAs [77,78]. The most recently identified type of non-coding RNA is circular RNA (circRNA), which consists of a covalently closed RNA loop that lacks a polyadenylation tail at the 3’ end and a cap structure at the 5’ end. These characteristics make cirRNAs resistant to exonucleases, which gives them a much longer half-life than the messenger RNAs from which they originate [79]. The dysregulated expression of circRNAs has been implicated in several diseases, including cancer [80,81,82,83].

## 6. microRNAs

Various roles for miRNAs in the regulation of physiological processes such as cell differentiation, proliferation and metabolism, as well as in pathologies such as cancer, have been described [84]. miRNAs function as post-transcriptional regulators of mRNA targets by base pairing with partially complementary sites on the 3’ untranslated regions (3’UTR), leading to translational repression or mRNA degradation, when completely paired to the seed region binding site [85]. It has been predicted that around 60% of all protein-coding genes contain sequences that could potentially bind to miRNAs [86].

## 7. Long Non-Coding RNAs

Currently, only a few mechanisms of action have been thoroughly characterized for mammalian lncRNAs, demonstrating that these ncRNAs are functionally and structurally different. Compared to the levels of mRNAs, lncRNAs are lower and their expression is significantly more constrained to specific cell types. lncRNAs regulate different cellular processes including transcription, translation and protein–protein interactions.

The localization of lncRNAs within the cell is frequently a good indicator of their role in the regulation of biological processes. In the nucleus, lncRNAs can repress or activate mRNA transcription [87]. lncRNAs might also regulate splicing, through their interaction with SRSF1, 2, and 3 splicing factors, which dictate exon recognition, ensuring the accuracy of splicing and regulating the alternative splicing of a subset of pre-mRNAs [88]. Moreover, in the nucleus, some lncRNAs can epigenetically modulate gene expression by acting as guidance molecules to modify the chromatin, controlling its architecture and participating in the formation of protein complexes or interfering with protein–protein interactions [89]. In the cytoplasm, lncRNAs modulate mRNA stability or translation. lncRNAs can compete with other ncRNAs, such as miRNAs, acting as molecular sponges, sequestering miRNA away from target mRNA, thereby upregulating their targets [90]. Finally, in the cytoplasm the lncRNAs can influence cellular signaling cascades, modulating their biological effects [91].

## 8. Circular RNAs

CircRNAs can regulate transcription through their interaction with miRNAs and lncRNAs, or by acting as competing endogenous RNAs (ceRNAs) via sponging and sequestering miRNAs and RNA-binding proteins [92,93]. Nevertheless, since some circRNAs are translated into proteins [94], they do not fit the precise definition of ncRNAs.

With respect to their origin, there are four classes of circRNAs: exonic circRNAs (ecircRNA), circular intronic RNAs (ciRNAs), exon-intron circRNAs and intergenic circRNAs [92,95,96]. It has been stated that circRNAs are mainly created from back-spliced exons. In such a process, downstream 3′ splice donors are covalently linked to upstream 5′ splice acceptors in swap order [97]. However, the precise mechanisms that drive circRNA biogenesis remains unclear.

Some studies have identified that circRNAs regulate cancer metabolism predominantly by acting as sponges for miRNAs [98,99,100]. By indirect association, it has been speculated that the circRNAs participate in the metabolism of glutamine [101,102].

## 9. The Role of miRNAs in the Regulation of Glutaminolysis in Cancer

### 9.1. miR-103a-3p

**Figure 2:**
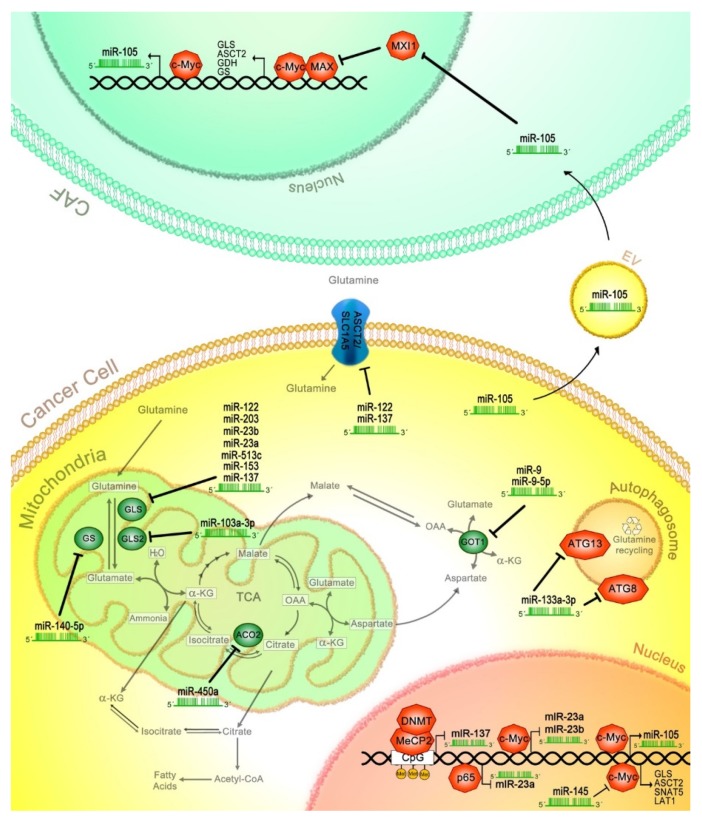
miRNAs affect glutaminolysis in cancer. Several miRNAs regulate proteins involved in transport or metabolism of glutamine. GLS is regulated by miR-122, -203, -23b, -23a, -513c, -153 and -137; while, miR-103-3p regulates GLS2. Moreover, miR-140-5p negatively regulates GS expression. GOT1 protein is posttranscriptional affected by miR-9 and -9-5p, while transporter ASCT2 translation is decreased via miR-122 and -137. Autophagy components such as autophagy related 13 and 8 (ATG13, ATG8) are suppressed through miR-133a-3p, blocking glutamine recycling. Additionally, the c-Myc transcription factor is inhibited by miR-145, affecting the expression of its targets, including GLS, ASCT, SNAT5, LAT1, miR-105, miR-23b and -23a. Furthermore, cancer cells secrete miR-105 in the extracellular vesicles (EV) reaching cancer-associated fibroblasts (CAF), where miR-105 promotes MAX interactor 1 (MXI1) mRNA degradation and subsequently induces the activation of glutaminolysis-related genes through the c-Myc/c-Myc associated factor X (MAX) transcriptional complex. Finally, p65 and methyl-CpG binding protein (MeCP2)/DNA methyltransferase (DNMT) negatively regulate miR-23a and miR-137, respectively, affecting the glutaminolysis pathway.

To date, several miRNAs have been implicated in the regulation of glutamine metabolism in cancer at different levels. In a gastric cancer cell line model, it was demonstrated that miR-103a-3p directly binds to the 3’UTR of the GLS2 mRNA, regulating its translation and, therefore, glutamine metabolism [103]. Interestingly, miR-103a-3p was found to be upregulated in gastric cancer inhibiting the glutamine pathway that positively regulates ferroptosis, which is a type of cell death characterized by the accumulation of reactive oxygen species produced by ion metabolism, as well as by the accumulation of lipid peroxidation products such as malondialdehyde. High expression of miRNA-103a-3p was associated with poor prognosis in advanced gastric cancer [104] (Figure 2).

### 9.2. miR-145

Li et al. (2019) [105] identified miR-145 as a regulator of glutamine metabolism, since it was found to be downregulated in ovarian cancer samples and derived cell lines, with a negative correlation to the GLS transcript. It was determined that miR-145 targets c-Myc mRNA, affecting GLS expression. The author showed that overexpression of miR-145 inhibits glutamine consumption, as well as the production of α-kG and cellular ATP. These results demonstrate that miR-145 can inhibit glutaminolysis in ovarian cancer cells through the c-Myc/GLS1 axis (Figure 2).

### 9.3. miR-450a

Moreover, in the late stages of epithelial ovarian cancer, miR-450a is found to be downregulated, compared to early cancer stages, acting as a tumor suppressor in this type of cancer. Interestingly, transcriptome analysis in A2780 ovarian cancer cells revealed that overexpression of miR-450a downregulated the genes involved in epithelial–mesenchymal transition (EMT), such as *VIM*, *TWIST1* and *SERPINE1*. Genes related to mitochondrial metabolism were also downregulated, including *ACO2*, *TIMMDC1*, *ATP5B* and *MT-ND2*. Comparable functions were also observed for miR-450b, affecting a similar set of transcripts. Furthermore, specific target analysis carried out in ovarian cancer cell lines revealed that ACO2 transcript is a potential direct target for miR-450a, which could explain the observed decrease in glutamate production and increase in glutamine consumption by miR-450a, since ACO2 converts citrate into isocitrate, regulating the Krebs cycle and glutaminolysis. Finally, overexpression of miR-450a induced a reduction in cell migration and invasion, while tumor growth was reduced in a murine xenograft model, suggesting that miR-450a acts as a tumor suppressor [106] (Figure 2).

### 9.4. miR-137

Luan et al. (2018) [107] and Luo et al. (2018) [108] demonstrated that miR-137 acts as a tumor suppressor, regulating two crucial elements of glutamine metabolism, GLS and SLC1A5 (ASCT2). When analyzing melanoma tissues, miR-137 expression was found to be decreased, while GLS was overexpressed in relation to the adjacent normal tissues. Multivariate analysis in melanoma patients demonstrated that low expression of miR-137 was an independent risk factor of overall survival. Additionally, miR-137 suppressed cell proliferation and glutamine catabolism by directly targeting GLS in in vitro and in vivo models [107]. When miR-137 was ectopically expressed in melanoma cells, it was found to bind to SLC1A5 mRNA, inhibiting SLC1A5 glutamine transporter translation. This event induced a decrease in glutamine uptake, which also prevented ferroptosis-related cell death, since glutaminolysis sensitizes to ferroptosis under certain conditions [108] (Figure 2).

Another study also demonstrated that the glutamine transporter ASCT2 is a target of miR-137 that regulates its expression, without affecting GLS [109]. When miR-137 was inhibited in colon epithelial cell lines, the levels of ASCT2 transporter increased. Dong et al. (2017) [109] reported that levels of miR-137 were inversely correlated with ASCT2 expression, demonstrating that low miR-137 expression and high levels of ASCT2 were characteristic of colorectal tumor specimens. However, in colorectal epithelial cells, when miR-137 was overexpressed, ASCT2 decreased. These data suggest that miR-137 plays a role in ASCT2 expression and glutamine metabolism. Similarly, when ASCT2 was depleted by miR-137 overexpression, glutamine consumption was inhibited, causing a decrement in ATP production and the levels of α-kG, suggesting that miR-137 downregulates glutamine influx in this type of cancer. Afterwards, nude mice were xenografted with shASCT2-treated HCT116 colorectal epithelial cancer cells, showing that tumor growth was reduced. Similar effects were observed with xenografted cells ectopically expressing miR-137. Interestingly, the effect of miR-137 was inhibited after the exogenous restoration of ASCT2. Furthermore, methyl-CpG binding protein (MeCP2) and DNA methyltransferase (DNMT) proteins epigenetically silence miR-137 expression through the methylation of CpG islands flanking its transcriptional start site. Consequently, ASCT2 expression is maintained permitting glutamine uptake. This was demonstrated with the inhibition of DNMT3B and MeCP2, where miR-137 expression was restored, while that of ASCT2 was reduced [109].

### 9.5. miR-9

The role of miR-9-5p in glutaminolysis has been described. It was demonstrated that miR-9-5p targets the 3’UTR of GOT mRNA, inducing its degradation, which affects glutaminolysis through a reduction in glutamate production. In pancreatic cancer samples, miR-9-5p was downregulated, which correlated with an overall shorter survival. In in vitro assays, the ectopic expression of miR-9-5p strongly repressed cell proliferation and invasion, and induced apoptosis [110]. Conversely, it has been reported that miR-9 is a negative regulator of ferroptosis, where glutaminolysis plays a crucial role. miR-9 binds to GOT1 3’UTR mRNA in melanoma cells, with a subsequent reduction in ferroptosis induced by Erastin and RAS-selective lethal (RSL3). Knockdown of miR-9 enhanced the expression of GOT1, allowing the production of high levels of α-kG from glutamate. In addition, miR-9 knockdown cells treated with glutaminolysis inhibitors did not respond to ferroptosis inductors. Additionally, anti-miR-9 treatment allows the production of high levels of α-kG through GOT activity. These findings indicate that miR-9 regulates glutaminolysis through GOT1 ablation, and is considered a negative regulator in melanoma cells [111] (Figure 2).

### 9.6. miR-105

It has been shown that MDA-MB-231 breast cancer cells secrete extracellular vesicles (EV) carrying high amounts of miR-105 [112]. When those EVs where administered to cancer-associated fibroblasts (CAF) derived from breast cancer patients, an upregulation in the transcription of c-Myc-dependent genes was observed, while MXI1 (a MAX-interacting protein) was downregulated. It is worth mentioning that c-Myc is a component of the heterodimeric MYC/c-Myc associated factor X (MAX) transcriptional factor, which is negatively regulated by MXI1. Later, it was shown that c-Myc and miR-105 increased glucose and glutamine uptake, potentiated when both elements were ectopically expressed in MCF10A non-tumoral breast cells. In this model, it was demonstrated that miR-105 targets MXI1, with the consequent activation of c-Myc. Interestingly, miR-105 contained in EV, in turn, induced the expression of endogenous miR-105 in CAFs and MCF10A cells, through the activation of c-Myc. Furthermore, a feedback loop was confirmed since c-Myc binds to the miR-105 promoter, inducing its transcription. Moreover, breast cancer cells that overexpress c-Myc tend to secrete higher levels of miR-105 in EV compared to non-cancerous cells. These results demonstrate the loop activation of Myc–miR-105–Myc. Therefore, high c-Myc activity allows for the high secretion of miR-105-containing EVs, which activate c-Myc signaling in the surrounding stromal cells, which consequently feedback to cancer cells, increasing the catabolism of glucose and glutamine. In those CAF EV/miR-105-educated cells, glutaminolysis elements were upregulated, including GLS and glutamine transporter SLC1A5, as well as GS and GDH. Moreover, it was described that reprogrammed CAFs increase glutamine and glutamate secretion to fuel glutamine-addicted cancer cells. Finally, in orthotopically transplanted patient-derived breast cancer cells, it was demonstrated that co-transplanted CAF EV/miR-105-educated cells significantly enhanced tumor growth, compared to those co-transplanted with CAF EV cells treated with anti-miR-105. Moreover, tumors exposed to CAF EV/miR-105 exhibited high levels of biosynthesis metabolites, such as amino acids, nucleotides, NADH and glutathione [112] (Figure 2).

### 9.7. miR-153

In glioblastoma tissue and glioblastoma cell lines, miR-153 is significantly downregulated. Glioblastoma cells ectopically expressing miR-153 showed a reduction in proliferation, an increase in apoptotic levels, restrictive glutamine utilization and glutamate generation in comparison to control cells. Since GLS is a target of miR-153, when GLS was knocked down, microRNA-153 was not able to reduce glutamine metabolism. Nevertheless, when GLS was ectopically overexpressed, the effect of miR-153 on GLS was abrogated, due to the absence of a 3’UTR in the exogenous GLS transcript. Interestingly, a negative correlation between GLS and miR-153 expression was observed in samples of glioblastoma, suggesting that miR-153 regulates glutamine metabolism in this type of cancer [113].

### 9.8. miR-513c

Conversely, in neuroblastoma, miR-513c was found to be downregulated in both cancer biopsies and cell lines. The restoration of miR-513c, in a neuroblastoma cellular model, showed a suppressive effect on proliferation, migration and invasion. This effect was attributed to the decrease in GLS mRNA, since it is a direct target of miR-513c. These findings suggest that miR-513c may act as a tumor suppressor and deserves to be studied for consideration in neuroblastoma treatment [114] (Figure 2).

### 9.9. miR-23a/b

It was determined that miR-23a and miR-23b target the 3’UTR of GLS, inhibiting its translation. It was shown that miR-23a and -23b are transcriptionally repressed by c-Myc in P-493 B lymphoma and PC3 prostate cancer cells, leading to the stabilization of miR-23a and -23b targets, such as GLS. As a consequence, glutamine metabolism is enhanced, supporting proliferation in c-Myc-expressing cells [46] (Figure 2).

In a model of human leukemic Jurkat cells, it was found that the transcriptional factor NF-kB p65 subunit binds to the miR-23a promoter, inhibiting its expression in the presence of glutamine. In consequence, GLS protein levels increase, which facilitate glutamine consumption and cellular adaptation to the metabolic environment [115] (Figure 2).

### 9.10. miR-203

Chang et al. (2017) [116] reported a negative correlation between GLS and miR-203 expression, where miR-203 is downregulated and GLS transcripts are upregulated in malignant melanoma (MM) biopsies. GLS mRNA is targeted by miR-203, promoting a decrease in glutamine uptake in MM cells. A lower expression of miR-203 was observed in temozolomide (TMZ)-resistant MM cells, compared with parental cells. Therefore, when GLS was knocked down through miR-203, the inhibition of glutamine uptake was observed, sensitizing MM cancer cells to TMZ chemotherapy [116] (Figure 2).

### 9.11. miR-133a-3p

Zhang et al. (2018) [117] identified miR-133a-3p as a tumor suppressor in gastric cancer cells. According to an analysis performed using The Cancer Genome Atlas (TCGA) database, the downregulation of miR-133a-3p was observed in gastric cancer samples as well as in gastric cancer-derived cell lines. It was determined that the overexpression of miR-133a-3p significantly reduced the proliferation rate and the number and size of colonies compared with a negative control group, where the ability of gastric cancer cells to migrate and invade was suppressed, and also the growth of gastric cancer organoids was significantly blocked. Additionally, it was demonstrated that miR-133a-3p acts via blocking the glutaminolysis pathway by binding to the 3’UTR region of gamma-aminobutyric acid receptor-associated protein-like 1 (GABARAPL1) and ATG13 that are involved in autophagy induction and vesicle nucleation. It is important to consider that, through the autophagy process, gastric cancer cells recycle glutamine for glutaminolysis to promote survival and metastasis. Therefore, it was confirmed that the malignant behavior of gastric cancer cells was suppressed by blocking glutaminolysis through the inhibition of autophagy by miR-133a-3p [117] (Figure 2).

### 9.12. miR-140-5p

Another miRNA implicated in the regulation of the glutaminolysis pathway is miR-140-5p, which binds to GS (GLUL) mRNA, regulating its expression. It was demonstrated that GS transcript and protein are upregulated in high-grade (III-IV) glioma cells. It is worth remembering that GS synthesizes glutamine from glutamate and ammonia, a process that is altered by miR-140-5p. When GS was knocked down by miR-140-5p, the inhibition of proliferation, migration and invasion was observed. These data suggest that GS participates in malignant glioma progression. Besides, miR-140-5p repressed proliferation, migration and invasion through GS downregulation, supporting a role for this miRNA as a negative regulator of glioma progression [118] (Figure 2).

### 9.13. miR-122

Finally, liver-specific miR-122 has been identified as a tumor suppressor through its effect on glutamine metabolism. Sengupta et al. (2020) [119] reported high glutamate and glutamine levels, as well as other intermediate metabolites of the TCA cycle, in the liver of a miR-122 knockout mice model. In this model, the expression and protein levels of GLS also increased, evidencing the role of miR-122 in the control of glutaminolysis. Besides, when miR-122 was overexpressed in the glutamine-dependent hepatocellular cancer cell line EC4, the attenuation of glutaminolysis was observed with the suppression of GLS activity, while gluconeogenesis increased. Interestingly, argonaute-crosslinking and immunoprecipitation assay (AGO-CLIP) identified the seed sequences of miR-122 in the 3’UTR of GLS mRNA. Moreover, microarray data analysis of miR-122 knockout mice revealed an increase in SLC1A5 (ASCT2) transporter expression, suggesting that miR-122 downregulates this glutamine transporter. These data were later confirmed using a luciferase assay, where miR-122 putative binding sequences in 3’-UTRs of GLS and SLC1A5 were identified as direct targets. Then, using Liver Hepatocellular Carcinoma database of The Cancer Genome Atlas (TCGA-LIHC), the authors demonstrated an inverse correlation between a high expression of GLS and SLC1A5 with low levels of miR-122 and related this to poor prognosis in high-grade primary HCC tumors [119] (Figure 2).

## 10. The Role of lncRNAs in the Regulation of Glutaminolysis in Cancer

The lncRNAs are commonly found to be altered in several types of cancer, affecting different hallmarks, including metabolic reprogramming. lncRNAs have been implicated in glutamine uptake, an important energy source of the cancer cell that maintains survival and proliferation, although the precise mechanisms of lncRNAs controlling this cellular process remain unclear [120].

### 10.1. lncRNA TUG1

**Figure 3:**
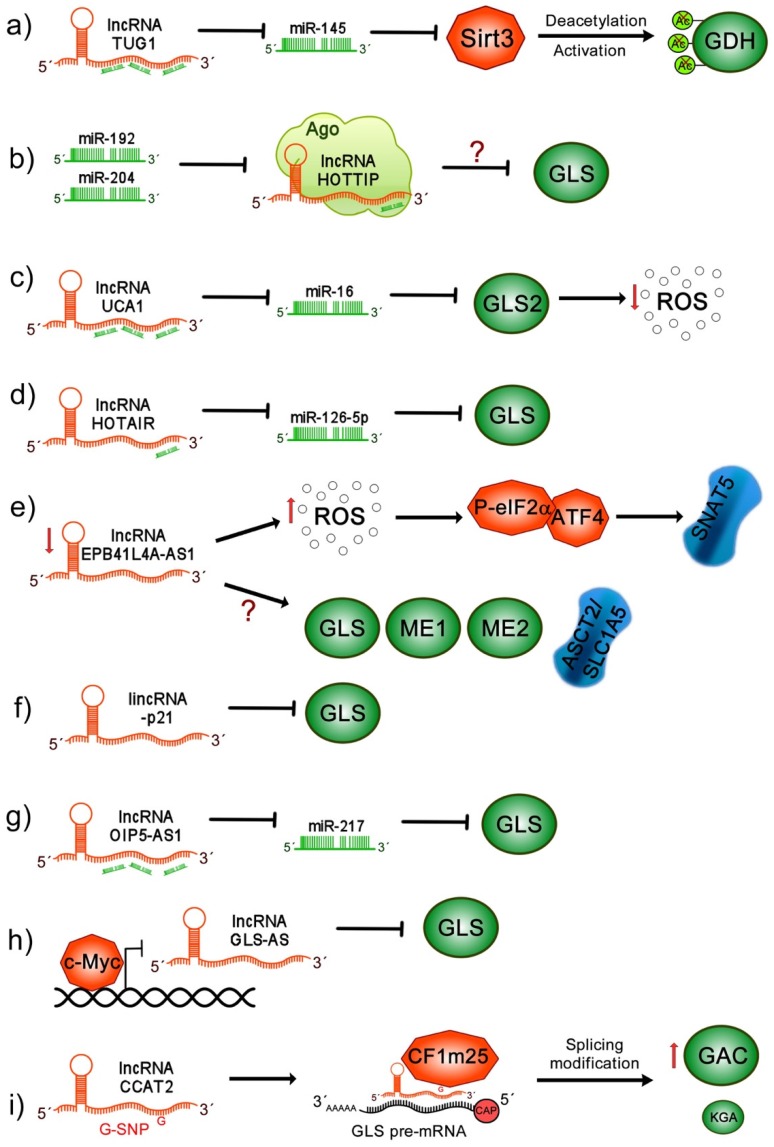
lncRNAs affect glutaminolysis pathway in cancer. (**a**) lncRNA taurine upregulated gene 1 (TUG1) acts as an miR-145 sponge, preventing Sirt3 mRNA degradation, which promotes glutamate dehydrogenase (GDH) deacetylation and its consequent activation; (**b**) miR-192 and miR-204 induce the suppression of lncRNA homeobox A (HOXA) distal transcript antisense RNA (HOTTIP) at the posttranscriptional level through Argonaute 2, inhibiting GLS expression; (**c**) lncRNA urothelial carcinoma associated 1 (UCA1) acts as sponge for miR-16, impairing the canonical binding of miR-16 to GLS2, promoting upregulation of GLS impacting in ROS reduction; (**d**) lncRNA homeobox (HOX) transcript antisense intergenic RNA (HOTAIR) functions as a ceRNA for miR-126-5p, modulating GLS expression; (**e**) low levels of lncRNA erythrocyte membrane protein band 4.1 like 4A (EPB41L4A)-AS1 induces high levels of reactive oxygen species (ROS), activation of P-eIF2α/ATF4 complex and overexpression of SNAT5 transporter. Moreover, through unknown mechanisms, EPB41L4A-AS1 leads an increase in ASCT2, GLS and ME1/2, leading to the increase in glutamine consumption; (**f**) long intergenic non-coding RNA p21 (lincRNA-p21) decreases GLS transcript and protein levels; (**g**) lncRNA opa-interacting protein 5 antisense transcript 1 (OIP5-AS1) acts as an miR-217 sponge upregulating GLS expression, contributing to the activation of glutaminolysis; (**h**) c-Myc inhibits lncRNA GLS-AS transcription, allowing the GLS stabilization; however, when lncRNA antisense lncRNA of glutaminase (GLS-AS) is expressed, mGSL is inhibited through the Adenosine Deaminase RNA Specific (ADAR)/dicer-dependent RNA interference; (**i**) lncRNA colon cancer associated transcript 2 (CCAT2) G allele binds to the CFIm25 subunit that interacts with GLS pre-mRNA and allows its alternative splicing, favoring the expression of glutaminase C (GAC) rather than kidney-type glutaminase (KGA), both GLS isoforms.

It has been shown that lncRNA taurine upregulated gene 1 (TUG1) expression is augmented in intrahepatic cholangiocarcinoma (ICC) samples, correlating with poor prognosis and adverse clinical pathological outcomes. Interestingly, it was found that TUG1 acts as an miR-145 sponge, preventing Sirtuin 3 (Sirt3) mRNA degradation [121]. Sirt3 is a mitochondrial protein that induces GDH deacetylation, increasing its activity [122]. Therefore, when the ablation of Sirt3 mRNA by miR-145 is prevented via TUG1, GDH is activated. This was demonstrated through TUG1 knock down in the ICC cell line, where glutamine consumption, α-kG production and ATP production were severely affected [121] (Figure 3).

### 10.2. lncRNA HOTTIP

Furthermore, the participation of lncRNA homeobox A (HOXA) distal transcript antisense RNA (HOTTIP) in glutaminolysis was studied in HCC cell lines. It was demonstrated that miR-192 and miR-204 induce the suppression of lncRNA HOTTIP at a posttranscriptional level through the Argonaute 2-mediated RNA interference pathway. When HOTTIP was silenced by miR-192 and miR-204, the significant suppression of cell viability was observed. In contrast, when mir-192 and miR-204 were antagonized, HOTTIP degradation was avoided, provoking an increase in cell proliferation [123]. In this study, GLS was identified as a potential downstream target of the miR-192/-204-HOTTIP axis that could interrupt glutaminolysis in this HCC model. Interestingly, in HCC samples, the expression of HOTTIP was upregulated, while miR-192 and miR-204 levels were downregulated, evidencing a clear negative correlation between them. Additionally, the dysregulation of the three ncRNAs was associated with poor overall survival of HCC patients [123] (Figure 3).

### 10.3. lncRNA UCA1

Moreover, it was shown that the lncRNA urothelial carcinoma associated 1 (UCA1) participates in the metabolic reprogramming of bladder cancer. The overexpression of UCA1 positively correlates with the upregulation of GLS mRNA and protein levels impacting in ROS reduction and mitochondrial glutaminolysis induction. This molecular mechanism can be explained since UCA1 acts as sponge for miR-16, impairing the canonical binding of miR-16 to the 3’UTR of GLS2 mRNA, thus avoiding its degradation [124] (Figure 3).

### 10.4. lncRNA HOTAIR

Homeobox (HOX) transcript antisense intergenic RNA (HOTAIR) has also been associated with the regulation of glutaminolysis in cancer. It has been found to be aberrantly upregulated in glioma samples. It was shown that HOTAIR modulates GLS expression by functioning as a competing endogenous RNA (ceRNA) for miR-126-5p, since this miRNA directly targets GLS mRNA, increasing glioma glutamine metabolism [125] (Figure 3).

### 10.5. lncRNA EPB41L4A-AS1

Afterwards, it was shown that the lncRNA EPB41L4A-AS1 is a p53 and peroxisome proliferator-activated receptor gamma coactivator 1-alpha (PGC-1α) inducible gene, and its low expression and deletion is frequently found in many human cancers, associated with poor prognosis. It was demonstrated that the depletion of erythrocyte membrane protein band 4.1 like 4A (EPB41L4A)-AS1 increases aerobic glycolysis and glutamine metabolism. The ectopic expression of EPB41L4A-AS1 induced a reduction in intercellular glutamate and α-kG levels. Similarly, in HeLa and HepG2 cells, it was observed that knock down of EPB41L4A-AS1 increases glutamine dependency. Interestingly, reactive oxygen species were augmented, inducing the transcriptional activation of P-eIF2α/ATF4; as a consequence, the overexpression of the SNAT5 (SN2) transporter was induced, leading to an increase in glutamine consumption. Moreover, it was also demonstrated that the absence of EPB41L4A-AS1 upregulates glutaminolysis-related members such as ASCT2, GLS, ME1 and ME2 [126] (Figure 3).

### 10.6. lincRNA-p21

Another lncRNA that is able to regulate glutamine catabolism is the long intergenic non-coding RNA p21 (lincRNA-p21), which has been found to be downregulated in cancer. It was demonstrated in bladder cancer cells that lincRNA-p21 exogenous expression reduced cellular growth and proliferation. In contrast, when lincRNA-p21 was silenced, the opposite effect was observed. Moreover, in lincRNA-p21-overexpressing cells a decrease in GLS transcripts and proteins was observed, with a subsequent reduction in intracellular levels of glutamate and α-kG. Interestingly, GLS overexpression in lincRNA-p21 knockdown cells was able to restore glutamine catabolism. This study suggests that lincRNA-p21 may act as a tumor suppressor through the regulation of glutamine catabolism, which depends on GLS, although the implicated mechanisms remain unknown [127] (Figure 3).

### 10.7. lncRNA OIP5-AS1

The role of lncRNA opa-interacting protein 5 antisense transcript 1 (OIP5-AS1) has been described in melanoma tumors, where it has been found to be significantly overexpressed. Additionally, the high expression of OIP5-AS1 was identified as an independent risk factor for the poor survival of patients with melanoma. Interestingly, the knockdown of OIP5-AS1 in A375 and SK-MEL-1 melanoma cells reduces proliferation and glutamine consumption. Additionally, glutamate and α-kG levels and ATP generation were also suppressed. It is proposed that OIP5-AS1 acts as an miR-217 sponge to upregulate GLS expression, since GLS is a specific target of miR-217. These results indicate that OIP5-AS1 may contribute to the malignant progression of melanoma-upregulating glutaminolysis [128] (Figure 3).

### 10.8. lncRNA GLS-AS

Additionally, it was found that the nuclear-enriched antisense lncRNA of glutaminase (GLS-AS) is involved in pancreatic cancer metabolism, where GLS-AS expression is downregulated and associated with short overall survival. In in vivo and in vitro models of pancreatic cancer cells with GLS-AS silencing, an increase in proliferation and invasion was observed. Interestingly, it was reported that GLS-AS inhibited the expression of GLS at the posttranscriptional level via the ADAR/dicer-dependent RNA interference. When cells were glucose and glutamine-deprived, GLS-AS was down expressed, while GLS mRNA and protein were upregulated, indicating that the deregulation of GLS-AS and GLS can be partially attributed to nutrient starvation stress. Moreover, it was demonstrated that c-Myc binds to the GLS-AS promoter and transcriptionally inhibits GLS-AS, which is exacerbated during nutrient deprivation. In contrast, c-Myc knockdown, during glucose and glutamine deprivation, increases GLS-AS expression. Reciprocal feedback was also demonstrated, where GLS-AS overexpression decreased c-Myc protein levels in a proteasome-dependent manner and also inhibited GLS expression. In contrast, GLS overexpression stabilized c-Myc during nutrient stress. Finally, the exogenous expression of GLS-AS reduced the proliferation and invasion of pancreatic cancer cells by impairing the c-Myc/GLS pathway [129] (Figure 3).

### 10.9. lncRNA CCAT2

It has been determined that lncRNA colon cancer-associated transcript 2 (CCAT2) participates in glutaminolysis. The overexpression of CCAT2 in colon cancer cells induces an increment of intra and extracellular glutamate, correlating with the high activity of GLS; however, glutamine consumption was not affected. Previously, it was shown that the *CCAT2* gene located at the 8q24 region harbors a cancer risk-associated rs6983267 single nucleotide polymorphism (SNP) (a change of T for G), of which the two known alleles have been shown to render distinct risks for colorectal cancer. For instance, the CCAT2 G allele is related to colorectal cancer predisposition. In HCT116 cells overexpressing CCAT2 G or T allele, it was observed that there were higher secreted glutamate levels in both G and T alleles, while intracellular glutamate production was only observed with the CCAT2 G allele and exhibited a higher GLS activity. Moreover, it was shown that the CCAT2 G allele induced higher GLS isoform GAC mRNA and protein levels than those observed for the KGA isoform. The two GLS isoforms contain the same active site; nevertheless, the GAC isoform has a higher catalytic activity than the KGA isoform, with the former being more competent in the induction of intermediates of the TCA cycle. These data suggest that the CCAT2 G allele favors the alternative splicing of the GAC GLS isoform. Moreover, it was also demonstrated that CCAT2 binds to the Cleavage Factor I (CFIm) complex, where the G allele preferentially binds to the CFIm25 subunit, while the T allele binds to the CFIm68 subunit. The G allele of CCAT2 interacts with UGUA nucleotide sequences located at intron 14 of the GLS pre-mRNA, allowing for the alternative splicing of GLS, and mediating the production of the GAC isoform. Additionally, in a xenograft mouse model, it was shown that the GAC isoform induced higher levels of metastasis and invasion in colorectal cancer [130] (Figure 3).

## 11. Implications of CircRNAs in the Glutaminolysis Pathway in Cancer

### 11.1. circHECTD1

**Figure 4:**
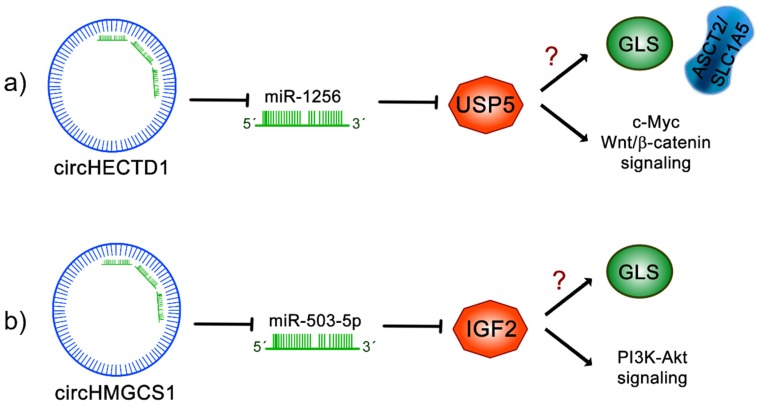
Regulation of cancer glutaminolysis pathways through circRNAs. (**a**) circRNA homologous to E6AP C terminus (HECT) domain E3 ubiquitin protein ligase 1 (circHECTD1) sponges miR-1256, leading to the stabilization of ubiquitin specific peptidase 5 (USP5) and inducing the activation of Wnt/β-catenin signaling and c-Myc signaling; interestingly, USP5 impacts the activation of glutaminolysis, leading to an increase in ASCT2 and GLS expression and, consequently, increased glutamine, glutamate and α-kG levels. (**b**) circRNA 3-hydroxy-3-methylglutaryl-CoA synthase 1 (circHMGCS1) inhibits miR-503-5p and has an impact on the stabilization of insulin-like growth factor 2 (IGF2), increasing the activation of phosphatidylinositol 3-kinase (PI3K-Akt) signaling activity. circHMGCS1 increases GLS levels, activating the glutaminolysis pathway and glutamine uptake. The question mark (?) indicates unknown mechanisms.

Recently, it was demonstrated that circRNA homologous to E6AP C terminus (HECT) domain E3 ubiquitin protein ligase 1 (circHECTD1) expression is significantly increased in gastric cancer biopsies compared to peritumoral samples, correlating with poor overall survival [131]. Similarly, in gastric cancer cell lines, the expression of circHECTD1 was higher than in normal gastric mucosal epithelial cells, where circHECTD1 is mainly localized in the cytoplasm. In gastric cancer cells, the exogenous overexpression of circHECTD1 induces an increase in proliferation, migration and invasion; conversely, when circHECTD1 is knocked down, those effects are reduced. In addition, glutaminolysis metabolites including glutamine, glutamate and α-kG levels were affected, being increased in circHECTD1-overexpressing cells and decreased in circHECTD1-silenced cell lines. Concordantly, it was found that circHECTD1 increased the expression of ASCT2 and GLS, affecting the glutaminolysis pathway. Interactome analysis of circHECTD1 revealed that mir1256 binds to circHECTD1, which was further confirmed by luciferase and RNA immunoprecipitation assays, involving the participation of the Ago2 complex. Interestingly, an inverse correlation of circHECTD1 and miR-1256 expression was observed in gastric cancer biopsies. In addition, when circHECTD1 is exogenously overexpressed, a reduction in miR-1256 levels can be observed in gastric cancer cells. It was then demonstrated that circHECTD1 regulates glutaminolysis and cancer progression by sponging miR-1256. Interestingly, through a bioinformatic analysis, it was found that miR-1256 directly targets ubiquitin specific peptidase 5 (USP5) mRNA, affecting its expression. Nevertheless, since circHECTD1 sponges mirR-1256, the transcript of USP5 is stabilized, leading to the activation of Wnt/β-catenin and c-Myc signaling pathways. Moreover, in circHECTD1-ablated gastric cancer cells with ectopic overexpression of USP5, the restoration of glutamine, glutamate and α-kG levels occurred, demonstrating the important role of USP5 in the regulation of glutaminolysis. Finally, in mice xenografted with circHECTD1-overexpressed cells, there was an increase in tumor size. In addition, USP5 overexpression occurred while reduced miR-1256 levels were observed. These data strongly suggest that the circHECTD1/miR-1256/USP5 axis regulates gastric cancer progression through the activation of glutaminolysis, Wnt/β-catenin and c-Myc signaling pathways [131] (Figure 4).

### 11.2. circHMGCS1

In addition, it has been demonstrated that circRNA 3-hydroxy-3-methylglutaryl-CoA synthase 1 (circHMGCS1) promotes hepatoblastoma (HB) tumorigenesis by sponging tumor suppressor miR-503-5p, which, consequently, upregulates the IGF-PI3K-Akt signaling pathway, increasing glutamine metabolism [132]. An analysis of the expression profile of circRNAs in HB tissues, through circRNA sequencing, demonstrated that the levels of circHMGCS1 are found to be significantly elevated in HB tissues compared to normal tissue samples. Further analysis in HB cell lines HepG2 and HUH6 and HCC-derived cell lines showed that circHMGCS1 was mainly expressed in the cytoplasm, with a higher expression in HB cells. The comparison between normal and HB tissues proved to have diagnostic value for circHMGCS1 and the increased levels of circHMGCS1 in HB patients was associated with a poor prognosis. In order to understand its biological function, the back-spliced junction sequence of circHMGCS1 was targeted by siRNAs in HB cell lines, which specifically silenced circHMGCS1 but not the linear HMGCS1 species. This approach inhibited cell proliferation and induced apoptosis. Conversely, when circHMGCS1 was overexpressed, cell proliferation significantly increased. The participation of circHMGCS1 in tumorigenicity was evaluated in xenograft tumor assays, where it was shown that knockdown of circHMGCS1 in the HUH6 cell line decreased tumor size, supporting the oncogenic function of circHMGCS1 in vivo. Interestingly, a metabolomic study revealed that circHMGCS1 knockout importantly reduced the mRNA and protein levels of GLS. Moreover, in HB tissues, a positive correlation between circHMGCS1 and GLS mRNA and protein expression levels was found. All these results confirm that circHMGCS1 regulates glutaminlosysis in HB by increasing GLS expression. Additionally, the circHMGCS1 interactome analysis and further dual luciferase assays confirmed that miR-153-3p, mir-490-5p, miR-615-3p and miR-503-5p could bind to circHMGCS1, attributing the later with a sponge function. Completing this work, the authors demonstrated that, by sponging miR-503-5p, circHMGCS1 upregulated its target, IGF2. Since PI3K-Akt are downstream targets of IGF2/IGF1R signaling, these events led to an increase in PI3K-Akt signaling and, therefore, to the increased expression of GLS, thus confirming that circHMGCS1 promotes proliferation and survival in HB, partly by sponging miR-503-5p, which generates the activation of the IGF2/IGF1R-PI3K-Akt axis, promoting glutaminolysis [132] (Figure 4).

## 12. Conclusions

Glutaminolysis is an essential metabolic pathway for tumor growth and maintenance. The identification of elements and regulators of this pathway is an essential requirement for developing not only effective therapeutic strategies against cancer, but also to identify prognostic biomarkers that could have an impact on clinical outcomes. Recently, ncRNAs, including miRNAs, lncRNAs and circRNAs have been identified as controlling glutaminolysis. Several studies confirm the participation of those ncRNAs in cancer development and establishment, modifying critical hallmarks of cancer, such as metabolic reprogramming and, in particular, glutaminolysis. Therefore, new information on the involvement of these biomolecules in glutaminolysis will undoubtedly strengthen future approaches to cancer treatment.
